# A delayed presentation of homozygous protein C deficiency in a series of children: a report on two molecular defects

**DOI:** 10.1002/ccr3.699

**Published:** 2017-02-06

**Authors:** Abdullah A. Baothman, Enaam AlSobhi, Hassan A. Khayat, Raed E. Alsulami, Abdulaziz S. Alkahtani, Abdelraheem A. Al‐Thobyani, Yousef I. Marzouk, Mohammad A. Abdelaal

**Affiliations:** ^1^ Pediatric Hematology UnitKing Abdulaziz Medical City National Guard Health Affairs‐Western RegionJeddahSaudi Arabia; ^2^ College of MedicineKing Saud bin Abdulaziz University for Health SciencesJeddahSaudi Arabia; ^3^ Hematology DepartmentKing Abdulaziz Medical City National Guard Health Affairs‐Western RegionJeddahSaudi Arabia; ^4^ Pathology DepartmentKing Abdulaziz Medical City National Guard Health Affairs‐Western RegionJeddahSaudi Arabia

**Keywords:** Deficiency, genotype, homozygous, presentation, protein C, purpura fulminans

## Abstract

Pediatric emergency visits with purpura fulminans should raise the suspicion of hereditary homozygous protein C deficiency even beyond the neonatal age. The absence of this classical finding does not role the diagnosis out as atypical presentation with isolated intraocular bleeding was observed. Premarital counseling should be offered when family history suggests.

## Abstract

We aimed to analyze the clinical presentation, age at onset, genotype of pediatric patients diagnosed with homozygous protein C deficiency (HPCD), and to provide a literature review. Five children with HPCD were included. Data were collected retrospectively from medical charts and prospectively from clinical visits. The information gathered for the five children was then compared to 82 patients reviewed in literature from 1984 to 2014. Three females and two males comprised the study population (*n* = 5). We detected the mutation c.1163 C>T (Ala388Val) in three of Five cases and c.1297 G>A (Gly433Ser) in two of five. In all cases, purpura fulminans (PF) was atypically delayed with mean age of 14.8 months (11–16 months) at first occurrence. Only one case described in literature with PF beyond one year, and this has different mutation c.8514 G>A (Ala267Thr). Blindness was seen in four of five. Of the four cases developed blindness, two were found to have the rare congenital ophthalmic finding of Peters anomaly. One case developed intraocular bleeding shortly after birth preceding PF, which occurred 16 months later. In contrast, all reported incidents of ocular bleeding were noted to occur around the time of PF (17 of 82, 21%). We defined two molecular defects causing HPCD with atypical presentations of late‐onset PF and isolated intraocular bleeding. No previous reports have provided clinical data for these particular mutations. Weather these molecular defects contributed to this atypical expression is yet to be answered. The association between Peter's anomaly and HPCD has never been reported. This observation merits further study in a large center.

## Introduction

Protein C (PC), first described by Stenflo in 1976 1, is a vitamin K‐dependent anticoagulant glycoprotein synthesized by the liver as a proenzyme. It is activated to serine protease by binding to thrombin–thrombomodulin complex on the vascular endothelium 2. Upon activation, PC inhibits the propagation of thrombus formation by inactivating factor Va and VIIIa with the aid of protein S, calcium ions, and phospholipids. Another function of PC is to enhance fibrinolysis by neutralizing circulating plasminogen activator inhibitor. This in turn leads to increased activity of tissue plasminogen activator (t‐PA) and thereby accelerates thrombus degradation 2.

Protein C gene (PROC) is located on chromosome 2q13‐14 consisting of nine exons and encoding a 1795 bp‐mRNA. This mRNA contains the protein C‐coding region (from exon 2 to 9) and nontranslated region. Each exon in the coding region is responsible for encoding specific structural and\or functional elements of the protein 3. More than 370 mutations have been described and linked to PC deficiency 4. Congenital PC deficiency is a rare cause of hereditary thrombophilia implicated only in about 3–5% of all thromboembolic events 5. The association between PC deficiency and the propensity toward venous thrombosis was first made by Griffin on 1981 6.Two types of congenital PC deficiency exist: type 1 (quantitative), which accounts for the majority of cases, is characterized by both reduced antigenic level and function and type 2 (qualitative) has decreased function in proportion to the antigenic level. Congenital PC deficiency is inherited as autosomal dominant disorder with marked variability in the penetrance and phenotypic expression 3.

The clinical presentation of PC deficiency varies according to how many alleles are affected. Heterozygous individuals with PC levels around 50% of the reference range have increased risk for venous thrombosis as compared to healthy population. Those patients can be asymptomatic or might develop thromboembolic events in early adulthood. The estimated incidence of asymptomatic heterozygous PC deficiency in healthy population is one in 200–500, while the symptomatic form accounts for one in 20,000 7.

HPCD with very low or undetectable activity level typically manifests in the neonatal period with life‐threatening PF and disseminated intravascular coagulation involving eyes, kidneys and CNS 7. Ophthalmic manifestations in a form of vitreous hemorrhage, which is due to retinal vessel thrombosis, have been reported and could be the initial presentation 8. Neurological complications such as ischemic stroke and hydrocephaly resulting from cerebral vein thrombosis may occur in utero and can be diagnosed antenatally 9. It is worth noting that not all patients with HPCD will have a full‐blown clinical picture in the neonatal period. Some reports have described individuals who developed venous thrombosis beyond the first month of life implying that other factors need to be present for full penetrance 10, 11, 12, 13, 14, 15. The incidence of homozygous PC deficiency is quite rare with only one case in 500,000 living birth annually 16. Some reports have estimated the incidence to be as low as one per 4 million births 7.

Herein, we aim to determine the genotype of five children with HPCD and analyze clinical presentation, prognosis, and review other comparable related data in the literature.

## Methods

Clinical and laboratory data for five patients, two males and three females, diagnosed with HPCD were collected retrospectively through chart review and during office visits. All of them are Saudis with median age of two years (2–14 years). They are registered in the database of National Guard Health Affairs‐Western Region (NGHA‐WR), King Abdulaziz Medical City (KAMC), Jeddah, Saudi Arabia.

We searched the literature (Medline Database) using the keywords mentioned above. The variables collected include age at onset, gender, type of presentation, family history, consanguinity, laboratory data, and genetic defects. We included all the available articles investigating HPCD that provided at least the clinical presentation and\or prognosis of the cases they studied. Only papers published in English language during the period from 1984 to 2014 were included. Data for 82 patients with HPCD have been reviewed**.**

Protein C activity was measured by activating protein C present in the patient sample using a specific snake venom activator. The quantitative determination of protein C activity was performed by a chromatographic substrate‐based assay. The device used is SIEMENS Berichrom* protein C provided at NGHA‐WR laboratory facilities.

The genetic study was carried out by DNA extraction of patient's leukocytes after obtaining an informed consent. The PROC gene was then amplified by polymerase chain reaction (PCR). Products of PCR were then sequenced for both DNA strands of the entire coding region and the highly conserved exon–intron splice junction. The genetic studies were carried out in cooperation with the laboratories of *Centogene AG* and *Biocentia*, Germany.

## Results

Clinical presentation (Table 1) and laboratory data (Table 2) along with genetic analysis (Table 3) are provided separately.

**Table 1:** Patients’ characteristics

Case	Purpura fulminans		Eye manifestation		Intracranial manifestation		FH^a^	Molecular defect	Outcome	Other association
	Age (month)	Site	Age	Type	Age	Type				
1	16	Thighs, abdomen, chest, and face	1 day	CO	16 month^b^	ICH, fibrosis, atrophy	(+)	A388V	Blindness	Peter's anomaly
2	16	Thighs, abdomen, chest, and face	1 day	CO	16 month^b^	ICH, fibrosis, atrophy	(+)	A388V	Blindness	Peter's anomaly
3	16	Abdomen, gluteal, and perineum	1 day	RH, CO	1 day	ICH	(+)	A388V	Blindness	Situs inversus
4	15	Legs, back, hand, and scalp	–	–	–	–	(+)	G433S	No disability	–
5	11	Lower limbs	1 day	RD	–	–	(+)	G433S	Blindness	Hemangioma

CO, corneal opacity; RH, retinal hemorrhage; RD, retinal detachment; ICH, intracranial hemorrhage.

a FH: family history; for further details, please refer to text.

b On this age, the imaging study was carried out; the lesion is suspected to be present prior to this imaging study.

**Table 2:** Molecular defects in protein C gene sampled from five children with HPCD and their biological parents

Case	State	Nucleotide sequence	Amino acid sequence	Paternal data				Consanguinity
				Father		Mother		
				PCa (%)	State	PCa (%)	State	
1	Homozygous	*E9: c.1163 C>T*	Ala388Val	70	Heterozygous^a^	40	Heterozygous^a^	+
2	Homozygous	*E9: c.1163 C>T*	Ala388Val					+
3	Homozygous	*E9: c.1163 C>T*	Ala388Val	40	Heterozygous^a^	49	Heterozygous^a^	+
4	Homozygous	*E9: c.1297 G>A*	Gly433Ser	57	Heterozygous^a^	42	Heterozygous^a^	+
5	Homozygous	*E9: c.1297 G>A*	Gly433Ser	NR	Heterozygous^a^	NR	Heterozygous^a^	+

PCa, protein C activity; NR, not recorded.

a Heterozygous for the same mutation seen in his\her offspring.

**Table 3:** Laboratory data of five Saudi children with homozygous protein C deficiency at presentation

Case	PLT (*10^9)	PT (sec)	PTT (sec)	INR	Fibrinogen (g\L)	D‐dimer (mg\L FEU)	PC activity (%)
1	59	18	200	1.6	0.4	35.9	0.01
2	56	17	44	1.4	0.4	35.8	<1
3	73	15	45	1.4	0.6	6.4	0
4	180	15	50	1.3	2.8	15	1
5	222	16	45	1.3	0.6	0.6	0

Normal values: PLT = 150–450 *10^9, INR = 0.9–1.2, PT = 11–17 sec, PTT = 28–42 sec, fibrinogen = 0.5–4 g\L, D‐dimer = <0.5, ESR = 0–10.

Two male twins and three unrelated females (*n* = 5) comprised the study population. Antenatal diagnosis of HPCD was not considered. All were born to consanguineous mates with heterozygous PC deficiency. Other siblings and\or family members with PC deficiency, both symptomatic and asymptomatic, were identified in all cases (*n* = 5). Protein C activity in all cases was <1% (70–120%). Acquired causes for protein C deficiency have been rolled out in all cases (*n* = 5).

In terms of frequency, PF ranks first as this was noted in all cases (*n* = 5). It involved multiple body sites with mean age of 14.8 months (11–16 months) at onset. Intracranial bleeding was noted in less frequency than PF (*n* = 3). Intracranial atrophy and fibrosis were also noted (*n* = 2). The age of onset for these intracranial lesions is not accurately identified. Blindness early in life was a common outcome (*n* = 4). Of the four cases developed blindness, two were noted to have the rare ophthalmic congenital finding of Peters anomaly. One child (case 3) has been confirmed to have ocular bleeding shortly after birth. Corneal opacity was seen in three cases.

## Discussion

Purpura fulminans, a thrombotic episode involving the small veins within the subcutaneous fat, is the prototype of HPCD 17. This manifestation usually develops over pressure areas and classically seen in the first 24–48 h of life. A picture of DIC is usually present 17. Literature review of 82 patients with HPCD showed PF in 53 patients (65%). In the present study, we noted PF in all patients (Table 1).

A strong history of positive consanguinity was noted in all cases we studied in comparison with 44% of the cases reviewed.

Ocular involvement is common among individuals with inherited thrombophilia, including PC deficiency. Vitreous hemorrhage is the most common finding 17. The exact cause is unknown, although retinal vein thrombosis is the likely underlying mechanism 17. Corneal opacity as a leading ocular manifestation associated with HPCD was documented in previous reports 18, 19, 20, 21, 22, 23. Leukocoria was also reported 8, 17, 24. In the present study, we noted that blindness is a common outcome of HPCD presented in four of the five cases (Table 1). In comparison, 16 of 82 (20%) patients reviewed in literature developed blindness. The rare ophthalmic finding of Peters anomaly was seen in two of four cases who developed blindness. Whether visual loss is related to HPCD or to be caused by this anomaly is not clearly determined. One case developed retinal detachment compared to 10 of 82 patients (12%) reported in literature.

Intracranial hemorrhage was seen in three of our cases in comparison with eight of 82 (10%) patients reviewed**.**

A total of 372 different mutations in PROC gene have been identified worldwide and reported in the human gene mutation database (HGMD) up to August, 2016. Most of these mutations are of nonsense or missense type 4. Exon 9 is particularly significant for protein C function as it encodes the catalytic site and the binding site for FVa 3. In our study, genetic testing detected two homozygous mutations involving exon 9 of PROC gene. Both mutations led to amino acid substitutions involving the catalytic domain of protein C structure, a key functional site. This explains the undetectable value of protein C activity observed in all cases (Table 2). The first mutation we identified is c.1163 C>T (Ala388Val) [Accession number: CM930622], whereas the second one is c.1297G>A (Gly433Ser) [Accession number: CM951034]. Both genetic defects have been previously described in the literature, but no clinical data were provided for comparison [25, 26].

The clinical expression associated with these genotypic variants is notably unusual in terms of onset and types of symptoms. In this study, PF, which is typically a neonatal finding, was seen beyond the first year of life. Literature review revealed PF delayed beyond the neonatal period in (6 of 82, 11%) patients. However, none of them was associated with the same genetic defects 10, 12, 27, 28. Only one case was reviewed in the literature with PF beyond one year, and this was positive for c.8514 G>A (Ala267Thr) [Accession number: CM920595] mutation 29. Whether these mutations played a role in the etiology of delayed‐onset purpura fulminans is yet to be elucidated.

In our study, we identified three cases with c.1163 C>T (Ala388Val) defect. One had ocular hemorrhage shortly after birth resulted in visual loss. However, the same case developed PF 16 months later. This was opposed to 17 reported patients (21%) with different genotypes where ocular hemorrhage was discovered around the time of PF. The genetic basis underlying this isolated finding of early‐onset ocular hemorrhage is yet to be explained. The other two cases with this mutation developed blindness associated with Peters anomaly. Although this anomaly was linked to other clinical manifestations 30, the association with HPCD has never been reported.

A number of limitations have to be stated. Given the rarity of HPCD, the limited number of cases presented in this study is not statistically sufficient to draw out conclusions about the data observed. In addition, This study used only functional assay to qualify the deficiency of PC as the antigenic level assay was not available. Thus, classifying the patients according to types of PC deficiency was not considered.

In conclusion, we report two genetic defects in HPCD with the atypical presentations of delayed PF and isolated ocular bleeding. As no previous study did provide clinical data for these particular mutations, it is legitimate to ask whether all individuals positive for the same mutations would have similar atypical expressions. This question is yet to be answered.

The second observation we made is the association between Peters anomaly and HPCD, which has never been reported. Whether a genetic link is established between Peters anomaly and HPCD is not clearly determined. This observation merits further study in a large center.

Considering the rarity and potentially irreversible outcomes of HPCD, prompt recognition and timely intervention can highly impact on the prognosis. Premarital genetic counseling and prenatal screening are prudent choices for consanguineous mates and/or those with suggestive family history.

## Conflict of Interest

None declared.
